# GSTΠ stimulates caveolin-1-regulated polyamine uptake via actin remodeling

**DOI:** 10.18632/oncotarget.27192

**Published:** 2019-10-01

**Authors:** Takeshi Uemura, George Tsaprailis, Eugene W. Gerner

**Affiliations:** ^1^ Amine Pharma Research Institute, Chuo-ku, Chiba 260-0856, Japan; ^2^ Center for Toxicology, College of Pharmacy, Tucson, Arizona 85721, USA; ^3^ Cancer Prevention Pharmaceuticals, Tucson, Arizona 85718, USA

**Keywords:** polyamine, caveolin, GSTπ, transport, actin

## Abstract

Polyamines spermidine and spermine, and their diamine precursor putrescine, are essential for normal cellular functions in both pro- and eukaryotes. Cellular polyamine levels are regulated by biosynthesis, degradation and transport. Transport of dietary and luminal bacterial polyamines in gastrointestinal (GI) tissues plays a significant role in tissue polyamine homeostasis. We have reported that caveolin-1 play an inhibitory role in polyamine uptake in GI tissues. We investigated the mechanism of caveolin-1-regulated polyamine transport. We found that glutathione *S*-transferase Π(GSTΠ) was secreted from caveolin-1 knockdown cells and stimulated spermidine transport in human colon-derived HCT116 cells. GSTΠ secreted in the medium increased *S*-glutathionylated protein level in the plasma membrane fraction. Proteomic analysis revealed that actin was *S*-glutathionylated by GSTΠ. Immunofluorescence microscopy demonstrated that actin filaments around plasma membrane were *S*-glutathionylated in caveolin-1 knockdown cells. Inhibition of actin remodeling by jasplakinolide caused a decrease in polyamine uptake activity. These data support a model in which caveolin-1 negatively regulates polyamine uptake by inhibiting GSTΠ secretion, which stimulates actin remodeling and endocytosis.

## INTRODUCTION

Polyamines, spermidine and spermine, and their precursor putrescine are essential factors for normal cellular functions [1]. Polyamines can bind to anions such as DNA, RNA and ATP, and regulate their functions [2]. Recent studies demonstrated that polyamines enhanced the synthesis of several proteins at the level of translation [3–5]. Polyamines are also required for posttranslational modification of eukaryotic translation initiation factor 5A (eIF5A), which is implicated in translation elongation and termination [6]. Although polyamines are required for normal cellular functions, high concentrations of polyamines are toxic to cells [7]. The cellular polyamine levels are tightly regulated by biosynthesis, degradation and transport. The transport plays important roles in polyamine homeostasis in addition to *de novo* synthesis and degradation systems [8]. The major sources of exogenous polyamines come from diet and luminal bacteria [9].

We have identified amino acid transporter SLC3A2 as a polyamine transporter in colon cancer derived cells [10]. SLC3A2 associates with polyamine catabolic enzyme spermidine/spermine *N*^1^-acetyl transferase, SAT1, and catalyzes the export of acetylated polyamines by a polyamine/arginine exchange reaction. In certain conditions such as low intracellular polyamine levels, SLC3A2 catalyzes polyamine uptake [11]. We have also reported that caveolar endocytic mechanisms mediates polyamine uptake and caveolin-1 play an inhibitory role in polyamine uptake in gastrointestinal tissues and human colon-derived HCT116 cells [11, 12].

Caveolin-1 is a major structural protein of caveolae in nonmuscle cells and is implicated in endocytosis and signal transductions [13]. Caveolin-1 is also involved in protein trafficking and secretion [14, 15]. Our previous study has demonstrated that knockdown of caveolin-1 expression using anti-sense RNA increased polyamine uptake in HCT116 cells [12]. It is suggested that polyamines are associated with lipid raft and internalized via caveolar endocytic pathway, but a detailed mechanism of caveolin-1-regulated polyamine uptake is not yet elucidated.

In this report, we investigated the mechanism of caveolin-1-regulated polyamine uptake. We found that glutathione *S*-transferase π (GSTπ) was secreted from caveolin-1 deficient cells and stimulated polyamine uptake in human colon-derived cells.

## RESULTS

### GSTπ secreted from caveolin-1 knockdown cells stimulates polyamine uptake

**Figure 1:**
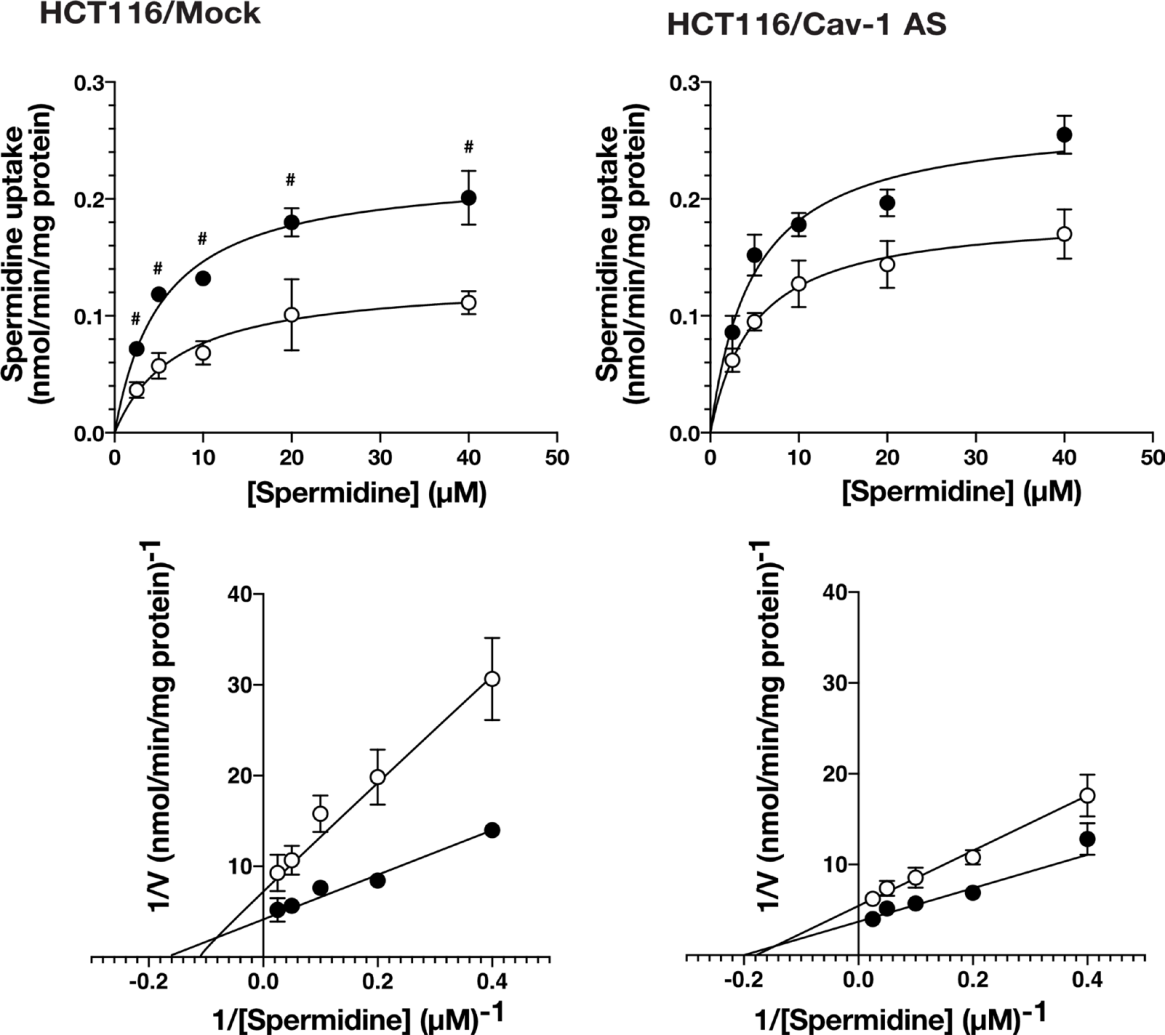
The effect of culture medium on spermidine uptake activity. HCT116/Mock and caveolin-1 knockdown cells HCT116/Cav-1 AS were cultured in the control supernatant (CS, white circles) or mutant supernatant (MS, black circles) for 2 days. Spermidine uptake activity was measured by incubating cells with radiolabeled spermidine for 5min as described in MATERIALS AND METHODS section. Values are mean ± SD of triplicate determinations. ^#^, *p* < 0.01. against HCT116/Mock cultured in CS. The corresponding double reciprocal plots are shown in bottom panels.

**Figure 2:**
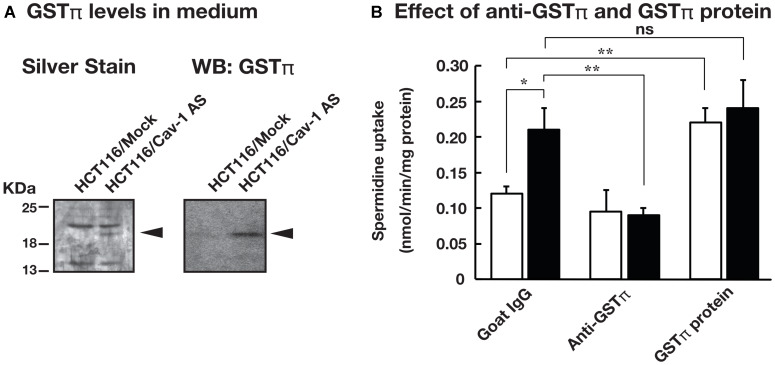
GSTπ was highly expressed in the culture medium of HCT116/Cav-1 AS cells and stimulated spermidine uptake. (**A**) HCT116/Mock and HCT116/Cav-1 AS cells were cultured in the serum-free Opti-MEM I for 2 days. Proteins in the medium were separated in 12% SDS-PAGE and stained as described in MATERIALS AND METHODS section. An arrowhead indicates the band highly expressed in the HCT116/Cav-1 AS culture medium. GSTπ protein was detected by western blotting using an anti-GSTπ antibody as described in MATERIALS AND METHODS section. (**B**) HCT116/Mock cells were cultured in control supernatant (white bar) or mutant supernatant (black bar) in the presence of 0.5 mg/mL anti-GSTπ antibody, normal goat IgG or 10 ng/mL purified GSTπ protein. Spermidine uptake activities were measured as described in MATERIALS AND METHODS section. Values are mean ± SD of triplicate determinations. ^*^, *p* < 0.05, ^**^, *p* < 0.01, ns, not significant.

We have reported that knockdown of caveolin-1 caused an increase in polyamine uptake [12]. Caveolin-1 is involved in endocytosis and protein secretion [14, 15]. We hypothesized that protein (s) secreted by caveolin-1 dependent mechanism can affect polyamine transport. To test this idea, we examined the effect of culture medium on the polyamine uptake activity using human colorectal carcinoma cell line HCT116 stably transfected with the expression vector containing caveolin-1-encoding cDNA in the antisense orientation (HCT116/Cav-1 AS) and empty vector transfected cells (HCT116/Mock). HCT116/Mock and HCT116/Cav-1 AS cells were cultured in the medium from HCT116/Mock (Control Supernatant, CS) and HCT116/Cav-1 AS (Mutant Supernatant, MS) culture, respectively, and spermidine uptake activity was measured. As shown in Figure 1, spermidine uptake activity was significantly increased when HCT116/Mock cells were cultured in MS. The *Vmax* value was about twice as high in HCT116/Mock cells cultured in MS, whereas *Km* values were not significantly affected (Table 1). Spermidine uptake activity was slightly increased in HCT116/Cav-1 AS cells when cultured in MS but the difference between CS was not significant (Table 1). The growth and viability were not significantly different in both cell lines (data not shown). These results suggested that the culture medium of HCT116/Cav-1 AS cells (MS) contained protein (s) that stimulated spermidine uptake. We analyzed proteins secreted into the medium by SDS-PAGE. HCT116/Mock and HCT116/Cav-1 AS cells were cultured in the serum-free Opti-MEM I. After 2 days culture, media were collected, filtered to remove cells, and proteins were resolved in a 12% polyacrylamide gel. As shown in Figure 2A, a 22 KDa band was found in HCT116/Cav-1 AS culture medium. This band was subjected to peptide sequencing by liquid chromatography followed by tandem mass spectrometry. The 22 KDa band was identified as glutathione *S*-transferase π (GSTπ) with 14 unique peptides comprising 60% sequence coverage (Supplementary Table 1). Western blot analysis of the medium using the anti-GSTπ antibody confirmed the expression of GSTπ in the medium of the HCT116/Cav-1 AS culture (Figure 2A). These results indicate that GSTπ secreted from HCT116/Cav-1 AS cells can stimulate spermidine uptake. To confirm that GSTπ stimulated polyamine uptake, we treated the culture medium with anti-GSTπ antibody. As shown in Figure 2B, mutant supernatant (MS) stimulated spermine uptake. The treatment of MS with anti-GSTπ antibody diminished this effect of the medium, whereas non-specific normal goat IgG did not. Addition of purified GSTπ protein to the medium increased spermidine uptake. These results confirmed that GSTπ in the medium stimulated spermidine uptake.

**Table 1:** Km and Vmax values for spermidine uptake

Cells	Medium^a^	*Km*^b^	*Vmax*^b^
		μM	nmol/min/mg protein
HCT116/Mock	CS	6.41 ± 1.04	0.12 ± 0.01
	MS	5.27 ± 0.93 ^ns^	0.22 ± 0.04 ^*^
HCT116/Cav-1 AS	CS	5.22 ± 0.27	0.19 ± 0.02 ^*^
	MS	4.81 ± 0.69 ^ns^	0.26 ± 0.10 ^ns^

^a^, Cells were cultured in the medium from HCT116/Mock (Control Supernatant, CS) or HCT116/Cav-1 AS (Mutant Supernatant, MS) culture.

^b^, Values are mean ± standard error of triplicate determinations.

^*^, *p* < 0.05 against HCT116/Mock cells cultured in CS ns, not significant against HCT116/Mock or HCT116/Cav-1 AS cells cultured in CS.

### Knockdown of caveolin-1 increases membrane associated GSTπ

**Figure 3:**
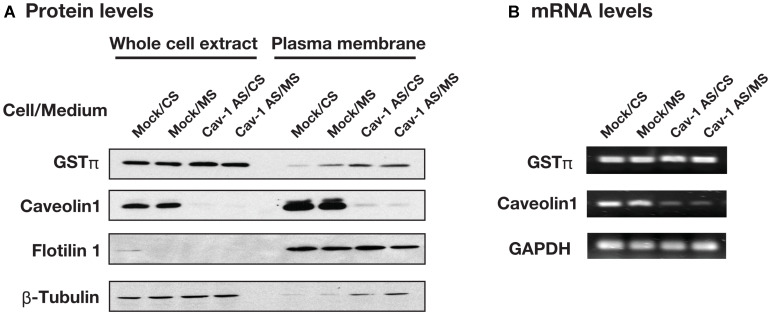
The effect of culture medium and caveolin-1 on the GSTπ expression. (**A**) HCT116/Mock and HCT116/Cav-1 AS cells were cultured in control supernatant (CS) or mutant supernatant (MS) and plasma membrane fractions were prepared. GSTπ, caveolin-1, flotilin 1 and β-tubulin in whole cell extract and plasma membrane fraction were detected by western blotting as described in MATERIALS AND METHODS section. (**B**) Total RNA was extracted from cells used in (A) and GSTπ, caveolin-1 and GAPDH mRNAs were detected by semi-quantitative RT-PCR as described in MATERIALS AND METHODS section.

**Figure 4:**
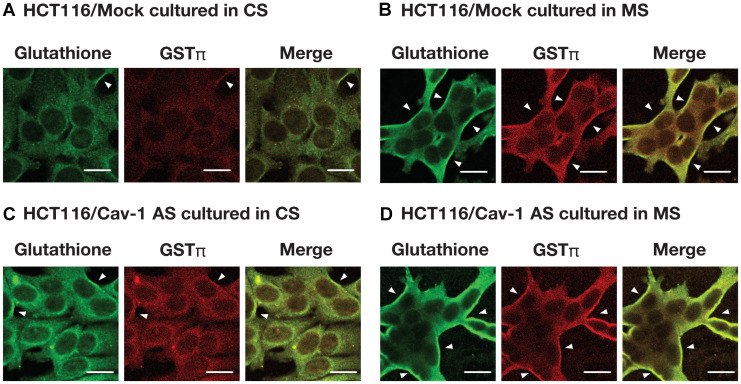
The effect of culture medium on *S*-glutathionylated protein and GSTπ levels. HCT116/Mock (**A**, **B**) and HCT116/Cav-1 AS cells (**C**, **D**) were cultured in the control supernatant CS (A, C) or mutant supernatant MS (B, D). After 24 hours, cells were fixed and stained as described in MATERIALS AND METHODS section. *S*-glutathionylated proteins (green), GSTπ (red) and merged images are shown. White arrowheads indicate the colocalization of *S*-glutathionylated proteins and GSTπ on the plasma membrane. Bar = 20 μm.

We examined the effect of caveolin-1 on GSTπ expression. As shown in Figure 3A, plasma membrane associated GSTπ was increased by mutant supernatant (MS) in HCT116/Mock cells. Plasma membrane associated GSTπ level was also high in HCT116/Cav-1 AS cells whereas the levels of GSTπ in whole cell extract were the same in all culture conditions. The separation of plasma membrane from whole cell extract was tested using flotilin 1 as a marker for caveolae containing plasma membrane and β-tubulin for a cytosolic fraction. Judged from the data, plasma membrane fraction was separated from whole cell extract. GSTπ mRNA levels were not changed in all culture conditions (Figure 3B). These results suggested that caveolin-1 negatively regulated the secretion of GSTπ. GSTπ catalyzes *S*-glutathionylation of cysteine residues in proteins under nitrosative stress conditions [16]. We next examined the effect of the culture medium on *S*-glutathionylated protein levels. HCT116/Mock and HCT116/Cav-1 AS cells were cultured in CS or MS and stained with anti-GSTπ and anti-glutathione antibodies. As indicated by white arrowheads in Figure 4, MS upregulated *S*-gulutathionylated protein and GSTπ levels on the cell surface. The shape of HCT116/Mock cells cultured in MS was slightly changed from rounded to elongated (Figure 4A and 4B). This may be caused by a *S*-glutathionylation of proteins. Experiments were repeated three times and the same results were obtained. This result indicated that secreted GSTπ catalyzed *S*-glutathionylation of protein (s) on the cell surface.

### Actin *S*-glutathionylated by GSTπ regulates polyamine uptake

**Figure 5:**
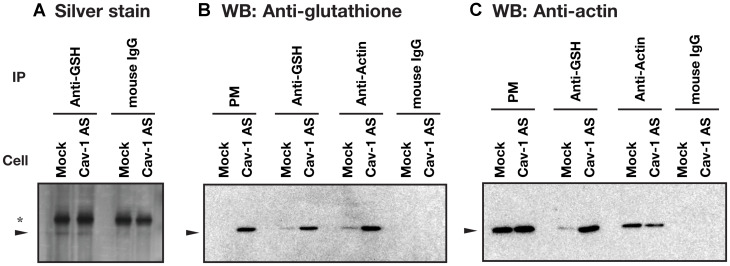
Identification of actin as a *S*-glutathionylated protein. Plasma membrane fractions prepared from HCT116/Mock and HCT116/Cav-1 AS cells were subjected to immunoprecipitation with indicated antibodies as described in MATERIALS AND METHODS section. Antibody-protein complex were analyzed by silver stain (**A**) and western blot analysis for glutathione (**B**) and β-actin (**C**) using TidyBlot: HRP conjugated western blot detection reagent. Arrowhead indicates 42 KDa protein. ^*^, a band from IgG. PM, plasma membrane.

**Figure 6:**
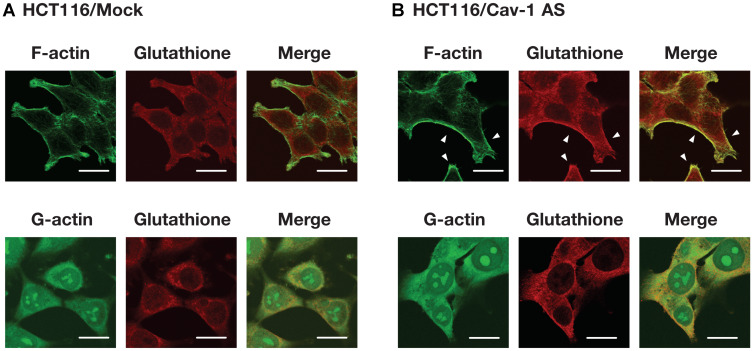
Cellular distribution of actin and *S*-glutathionylated protein. HCT116/Mock (**A**) and HCT116/Cav-1 AS (**B**) cells were cultured for 2 days, fixed and stained for F-actin, G-actin and glutathione as described in MATERIALS AND METHODS section. F- and G-actin (green), glutathione (red) and merged images were shown. White arrowheads indicate colocalization of fluorescence from F-actin and *S*-glutathionylated proteins. Bar = 20 μm.

**Figure 7:**
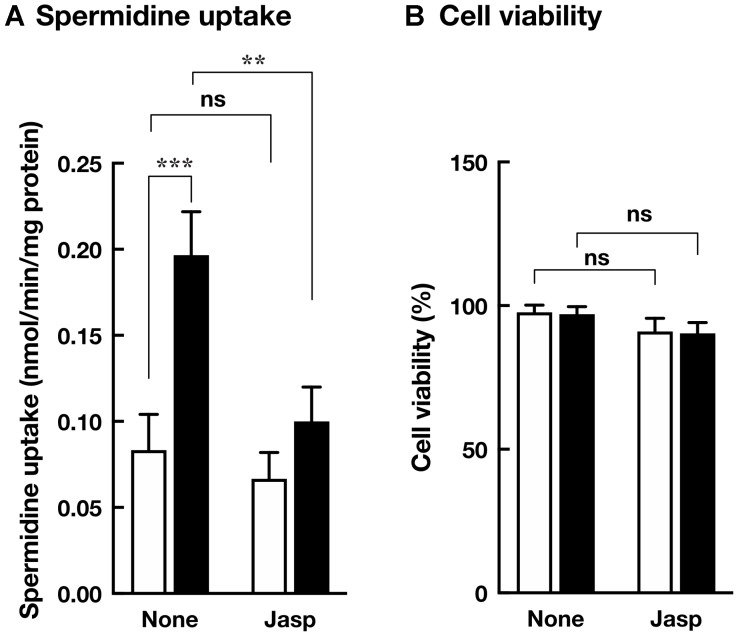
Effect of jasplakinolide on polyamine transport. (**A**) HCT116/Mock (white bar) and HCT116/Cav-1 AS cells (black bar) were cultured in the presence and absence of 100 nM jasplakinolide for 4 hours and washed with transport assay buffer. Spermidine uptake activities were measured as described in MATERIALS AND METHODS section. (**B**) The viability of cells cultured in the presence and absence of 100 nM jasplakinolide for 4 hours are counted. Values are mean ± SD of triplicate determinations. ^*^, *p* < 0.05, ^**^, *p* < 0.01, ns, not significant.

To identify *S*-glutathionylated protein, we performed immunoprecipitation using anti-glutathione antibody and plasma membrane fractions prepared from HCT116/Mock and HCT116/Cav-1 AS cells. As shown in Figure 5A, anti-glutathione antibody precipitated 42 KDa protein which was identified as β-actin with 15 unique peptides comprising 47% sequence coverage (Supplementary Information) by proteomic analysis. Western blot analysis demonstrated that protein precipitated with anti-glutathione antibody was β-actin, and β-actin was *S*-glutathionylated (Figure 5B and 5C). Immunofluorescence microscopy revealed that signal from *S*-glutathionylated protein was colocalized with F-actin on the cell surface area in HCT116/Cav-1 AS cells (Figure 6, white arrowheads). On the other hand, G-actin stained with DNase I did not colocalize with *S*-glutathionylated proteins (Figure 6). The experiment was repeated three times and the same results were obtained. We next examined the role of actin *S*-glutathionylation on polyamine uptake. It has been reported that *S*-glutathionylation of actin regulated actin filament formation by inhibiting actin polymerization [16, 17]. It was also reported that actin depolymerization induced caveolae-mediated endocytosis [18]. We hypothesized that *S*-glutathionylation of actin induced actin depolymerization and caveolar endocytosis. To test this idea, we treated cells with jasplakinolide, an inhibitor of actin depolymerization [19]. As shown in Figure 7A, jasplakinolide treatment inhibited spermidine uptake. In this experimental condition, jasplakinolide did not induce significant cell death (Figure 7B). Jasplakinolide was washed away before spermidine uptake assay so it was not likely the drug affected the binding of spermidine to the polyamine receptor. These data indicated that *S*-glutathionylation of actin by GSTπ induced actin depolymerization and endocytosis of polyamines.

## DISCUSSION

In this report, we investigated the mechanism of caveolin-1-regulated polyamine uptake. We found that culture medium of caveolin-1 knockdown cells contained GSTπ and increased *Vmax* for spermidine uptake without changing *Km* values (Table 1). These results indicated that GSTπ increased the rate of polyamine internalization but did not affect the affinity for polyamine recognition by cells. Proteomic analysis and an immunofluorescence microscope revealed that *S*-glutathionylation of actin on the cell surface area was increased in caveolin-1 knockdown cells. Reversible *S*-glutathionylation of actin was reported to regulate actin polymerization and actin filament formation by inducing structural change [20, 21]. It was suggested that *S*-glutathionylation of actin filaments induced actin depolymerization. Shen and Turner reported that actin depolymerization induced caveolae-mediated endocytosis [22]. Our data shown in Figure 7 indicated the inhibition of actin depolymerization by jasplakinolide reduced spermidine uptake activity. Our results demonstrate that caveolin-1 negatively regulates caveolar endocytosis dependent polyamine uptake by inhibiting GSTπ secretion, which stimulates actin depolymerization and endocytosis.

As for polyamine transport, Belting *et al.* reported that nitric oxide mediated a release of polyamines from glypican-1 in endosomes [23]. We have reported that nitric oxide synthase 2 (NOS2) was required for polyamine uptake in gastrointestinal tissues [11]. These reports suggest that the nitrosative stress is involved in the polyamines transport. In addition, the loss of caveolin-1 induces nitrosative stress [24]. As Townsend *et al*. showed, actin was *S*-glutathionylated by GSTπ under nitrosative stress condition [16]. Together with our results, it was suggested that nitrosative stress and *S*-glutathionylation could play important roles in polyamine uptake.

The role of caveolin-1 in GSTπ secretion is not yet elucidated. Recently Han and Zhu reported that caveolin-1 negatively regulated cell invasion through regulating expression and secretion of matrix metalloproteinase [25]. Yamaguchi *et al.* reported that the secretion of insulin-like growth factor binding protein-5 (IGFBP-5) was decreased by caveolin-1 [26]. These reports suggested that caveolin-1 could affect the expression and secretion of several proteins. It is possible that the secretion of GSTπ is regulated by the common mechanism underlying the secretion of these proteins. Our result indicated that caveolin-1 did not affect the expression level but altered the cellular distribution of GSTπ (Figure 3). The effect of 1α,25(OH)_2_ vitamin D_3_, an activated form of vitamin D_3_ on GSTπ secretion and calreticulin knockingdown experiment suggested that the secretion of GSTπ was not matrix vesicle dependent (data not shown). The inhibition of GSTπ secretion by 1α,25(OH)_2_ vitamin D_3_ was calreticulin independent (data not shown) and this suggested that cytosolic vitamin D receptor might regulate GSTπ secretion in a caveolin-1-regulated manner. Since knocking down caveolin-1 increases endocytosis rate, it is possible that increased GSTπ secretion in caveolin-1 knockdown cells involves altered endosomal sorting processes and vesicular recycling pathway due to the increased endocytosis and potential change of cellular localization of GSTπ. Further study is needed to clarify the molecular mechanisms of caveolin-regulated GSTπ secretion.

Our results suggest that high expression of GSTπ can cause an increase in polyamine uptake. A high expression of GSTπ is reported in human colorectal polyps and its expression level was correlated with the grade of adenoma [27]. In addition, it was reported that *N*-Myc upregulates GSTπ expression in neuroblastoma [28]. Since uptake of dietary and luminal bacterial polyamines significantly contributes to tissue polyamine levels and overcomes the effect of the polyamine biosynthesis inhibitor DFMO in mice model [29], a combination of polyamine biosynthesis inhibitor and uptake blocker is a potential strategy for cancer treatment and prevention. Indeed, it is reported that a combination of polyamine biosynthesis inhibitor and uptake blocker reduced tumor progression and prolongs survival in mouse models of neuroblastoma [30] as well as pancreatic cancer model mice [31]. Our study provides a novel target and strategy for cancer prevention and treatment using polyamine transport inhibitors in GSTπ expressing cells. For example, a GSTπ inhibitor such as 4-aryl-1,3,4-oxazolylium-5-olate [32] can be used for cancer prevention and treatment with a combination of DFMO. However, GSTs are involved in the detoxification of xenobiotics as well as oxidative stress [33]. Inhibition of GSTs may increase the risk of other disease such as brain stroke [34]. To maximize the therapeutic effect, the toxicity of the treatment should be carefully monitored. The effect of GSTπ inhibitor on polyamine transport and its therapeutic efficacy will be examined in the future.

## MATERIALS AND METHODS

### Cell culture

The human colorectal carcinoma cell lines HCT116, transfected with mock vector (HCT116/Mock) or caveolin-1 antisense (HCT116/Cav-1 AS), were a kind gift from Dr. B. Sloane and Dr. D. Cavallo-Medved [35]. The caveolin-1 was downregulated more than 80% in HCT116/Cav-1 AS cells compared to the TCH116/Mock cells. The growth and cell viability in both cell lines were the same under this culture condition. Cells were maintained in Dulbecco’s Modified Essential Medium (DMEM) supplemented with 10% fetal bovine serum (FBS), 1% penicillin/streptomycin (P/S) and 0.5 mg/mL puromycin. Cells were maintained in a humidified incubator at 37° C with 5% CO_2_. The viability of cells was monitored by staining with 0.2% trypan blue solution.

### Spermidine transport assay

Assays were performed as described previously [10] using [^3^H] spermidine (37 MBq/mmol, GE Healthcare) as a substrate. One million cells were cultured on a 6-well plate (greiner bio) for 2 day and the culture medium were collected, centrifuged and filtered through a 0.22 μm filter (Millipore). Another one million cells were cultured on a 6-well plate for 1 day and the culture medium were changed to the collected medium. Cells were cultured for another 2 days before spermidine transport assay. After 2 days, cells were washed with transport assay buffer containing 5 mM Hepes-NaOH, pH 7.4, 145 mM NaCl, 3 mM KCl, 1 mM CaCl_2_, 0.5 mM MgCl_2_ and 5 mM glucose and incubated in the same buffer for 5 min at 37° C. Spermidine uptake was started by the addition of [^3^H] spermidine. After 5 min incubation, cells were washed twice with ice-cold assay buffer containing 1 mM spermidine. Cells were lysed in 0.5 N NaOH and radioactivity was counted using a Beckman LS 5000TD scintillation counter. Total cellular protein was determined by the bicinchonic acid protein assay kit (Pierce). When the effect of the antibody was tested, anti-GSTπ antibody (abcam) and normal goat IgG were washed by diluting 1000-fold with serum-free DMEM and filtering through a microcon concentrator 3 centrifugal filters (amicon) three times to remove sodium azide. Cells were cultured with 0.5 mg/mL antibodies or 10 ng/mL of purified human GSTπ protein (Alpha Diagnostics International) for 24 hours and polyamine uptake was measured. For jasplakinolide treatment, cells were cultured with 100 nM jasplakinolide (Invitrogen) for 4 hours prior to the spermidine transport assay.

### Liquid chromatography coupled to tandem mass spectrometry (MS/MS) analysis

HCT116/Mock and HCT116/Cav-1 AS cells were cultured in the serum-free Opti-MEM I (GIBCO). After 2 days, media were collected, filtered through cellulose nitrate filter (pore size 0.2 mm, NALGENE) and incubated on ice with 5% trichloroacetic acid for 30 min. Proteins in media were precipitated by centrifugation at 16,000 × g for 30 min and then resolved on 12% SDS polyacrylamide gel and stained using ProteoSilver Plus Silver Stain Kit (Sigma). Bands highly expressed in HCT116/Cav-1 AS cell culture medium were excised and digested with trypsin for 16 hours (20). The extracted peptides from the gel following digestion were analyzed by a ThermoFinnigan LTQ ion trap mass spectrometer (San Jose, CA) equipped with a Michrom Paradigm MS4 HPLC (Auburn, CA) and a nanoelectrospray source as described previously (11). All spectra were searched against the ipi. HUMAN. v.3.27 protein database which, at the time of download, contained 67,528 protein entries (ftp://ftp.ebi.ac.uk/pub/databases/IPI/current/). MS/MS spectra were searched with Sequest and X!Tandem and results compiled using Scaffold as previously described [10].

### Preparation of plasma membrane fraction

A plasma membrane fraction was prepared by the method of Nishiumi and Ashida [36]. Cells were suspended in buffer A (50 mM Tris, pH 8.0, 0.1% Nonident P-40 (NP-40), 10 mg/mL aprotinin, 500 μM sodium orthovanidate, and 10 mg/mL phenylmethylsulfonyl fluoride) and passed three times through a 25-guage needle. The homogenate was centrifuged at 1,000 g for 10 min at 4° C, and the precipitate was suspended in NP-40 free buffer A, kept on ice for 10 min and centrifuged at 1,000 g for 10 min at 4° C. The precipitate was suspended in buffer A containing 1% NP-40, kept on ice for 1 hour and centrifuged at 16,000 g for 20 min at 4° C. The supernatant was collected as a plasma membrane fraction.

### Western blotting

Cells were washed with buffered saline and lysed in 10 mM Tris-HCl, pH 8.0 containing 10 mg/mL aprotinin, 500 μM sodium orthovanidate and 10 mg/mL phenylmethylsulfonyl fluoride, and used as whole cell lysate. Forty mg protein was separated on a 10% polyacrylamide gel. Proteins were transferred electrophoretically to a Hybound-C nitrocellulose membrane (Amersham, Arlington Heights, IL). Blots were blocked in 5% nonfat dry milk in tris buffered saline containing 0.1% Tween 20 (TBS-T) for 30 min at room temperature. GSTπ, *S*-glutathionylated proteins, caveolin-1, β-actin, β-tubulin, flotilin and calreticulin were detected by ECL Western Blotting Detection System (GE Healthcare) using anti-GSTπ (1:3000 dilution, Abcam), anti-glutathione (1:10000 dilution, Abcam), anti-caveolin-1 (1:10000 dilution, SANTA CRUZ BIOTECHNOLOGY), anti-β-actin (1:10000 dilution, Abcam), anti-β-tubulin (1:10000 dilution, SANTA CRUZ BIOTECHNOLOGY), anti-flotilin (1:1000 dilution, Cell Signaling) and anti-calreticulin (1:1000, abcam) as primary antibodies.

### Semiquantitative RT-PCR analysis

Total RNA was isolated using the Qiagen RNeasy Kit according to the manufacturer’s protocol. Two micrograms of total RNA were treated with RNase-free deoxyribonuclease I (Fermentas Life Science) and reverse transcribed into cDNA using the M-MuLV reverse transcriptase (Fermentas Life Science). The DNA fragments of GSTπ, caveolin-1 and glyceraldehyde 3-phosphate dehydrogenase (GAPDH) were amplified using primer sets of GSTP1-F (5′-TATTTCCCAGTTCGAGGCCG-3′) and GSTP1-R (5′-ATTGATGGGGAGGTTCACGTA-3′) for GSTπ, SLC3A2-F, CAV1-F (5′-TCAACCGCGACCCTAA ACACC-3′) and CAV1-R (5′-TGAAATAGCTCAGAAG AGACAT-3′) for caveolin-1, GAPDH-F (5′-TGGTATCGTGGAAGGACTCGTGGAAGGACTCATGAC-3′) and GAPDH-R (5′-AGAGTCCAGTGAGCTTCCCGTTCAGC-3′) for GAPDH.

### Indirect immunofluorescence microscopy

HCT116/Mock and HCT116/Cav-1 AS cells were cultured on cover slips, washed with buffered saline and fixed in 2% paraformaldehyde for 15 minutes at 37° C. Cells were then permeabilized with acetone for 30 seconds at −20° C, treated with 1% bovine serum albumin in PBS for 1 h at room temperature and incubated with primary antibody for 16 hours at 4° C. Cells were washed 5 times with the same buffer and incubated with fluorophore labeled secondary antibody for 16 hours at 4° C. Cellular F- or G-actin was stained using Alexa Fluor 488 conjugated phalloidin or DNase I (Invitrogen), respectively. After incubation, cells were washed 5 times and mounted in Prolong Gold Mounting Solution (Clontech). Fluorescence was visualized using a Nikon PCM2000 Confocal Microscope (Nikon) or Leica SP5 High-speed Spectral Confocal Microscope (Leica).

### Immunoprecipitation

Immunoprecipitation was performed as previously described [10] with modifications. One hundred mg of plasma membrane protein was incubated with anti-glutathione or anti-β-actin (Abcam) antibodies in IP buffer (50 mM Tris-HCl, pH 8.0, 120 mM NaCl, 0.5% NP-40) at 4° C for 16 h. Plasma membrane mixture were incubated with protein A agarose beads (SANTA CRUZ BIOTECHNOLOGY) for 16 h at 4° C and washed 5 times with IP buffer. Beads were suspended with 40 ml of SDS-sample buffer and boiled for 5 min. Proteins were separated on a 10% SDS-acrylamide gel, transferred to a Hybound-C nitrocellulose membrane and detected as described above using TidyBlot Western blot detection reagent: HRP (BioRad) to reduce the background from IgG. The protein that immunoprecipitated with anti-glutathione antibody was identified as descried above.

### Statistical analysis

Statistical analysis was performed using GraphPad Prism version 6.0d for Mac, GraphPad Software, La Jolla California USA, www.graphpad.com. Differences between two groups were compared using Student’s t-test. For comparison of multiple groups, one-way ANOVA followed by Tukey’s multiple comparisons test was used. A *p*-value < 0.05 was considered statistically significant.

The datasets generated during and/or analyzed during the current study are available from the corresponding author on reasonable request.

## SUPPLEMENTARY MATERIALS




