# Supplementary material for: GSTΠ stimulates caveolin-1-regulated polyamine uptake via actin remodeling
Source: Oncotarget. 2019 Oct 1;10(55):5713–23. doi: 10.18632/oncotarget.27192 (PMC6779281; doi:10.18632/oncotarget.27192)
Supplement: Supplementary file 1 [file oncotarget-10-5713-s001.pdf]

# GSTΠ stimulates caveolin-1-regulated polyamine uptake via actin remodeling

## SUPPLEMENTARY MATERIALS

**Supplementary Table 1:** See Supplementary Table 1
