# Supplementary material for: GSTΠ stimulates caveolin-1-regulated polyamine uptake via actin remodeling
Source: Oncotarget. 2019 Oct 1;10(55):5713–23. doi: 10.18632/oncotarget.27192 (PMC6779281; doi:10.18632/oncotarget.27192)
Supplement: Supplementary file 2 [file oncotarget-10-5713-s002.docx]

**Supplemental Table 1:**

| **Peptide Sequence** | **[M+H]+** | **Charge State** | **Sequest Xcorr** | **Sequest dCn** | **X!Tandem –log(e) value** |
| --- | --- | --- | --- | --- | --- |
| PFETLLSQNQGGK | 1419.16 | 2 | 4.49 | 0.274 | 0 |
| MLLADQGQSWK | 1278.96 | 2 | 3.96 | 0.347 | 5.92 |
| DQQEAALVDMVNDGVEDLR | 2119.03 | 3 | 4.76 | 0.386 | 0 |
| DQQEAALVDMVNDGVEDLR | 2117.18 | 2 | 4.41 | 0.44 | 15.8 |
| TVVYFPVR | 981.86 | 2 | 2.54 | 0.118 | 0 |
| AFLASPEYVNLPINGNGK | 1906.3 | 2 | 4.34 | 0.385 | 6.47 |
| AFLASPEYVNLPINGNGK | 1906.51 | 3 | 4.39 | 0.225 | 0 |
| ALPGQLKPFETLLSQNQGGK | 2127.28 | 2 | 4.37 | 0.515 | 8.85 |
| ALPGQLKPFETLLSQNQGGK | 2128.63 | 3 | 4.85 | 0.273 | 0 |
| EEVVTVETWQEGSLK | 1735.62 | 2 | 7 | 0.545 | 9.28 |
| FQDGDLTLYQSN | 1402.29 | 1 | 3.21 | 0.24 | 0 |
| FQDGDLTLYQSNTILR | 1884.26 | 2 | 5.97 | 0.497 | 12.5 |
| ETLLSQNQGGK | 1175.96 | 2 | 2.6 | 0.316 | 0 |
| LKPFETLLSQNQGGK | 1661.42 | 2 | 4.76 | 0.378 | 0 |
| PPYTVVYFPVR | 1337.56 | 1 | 2.58 | 0.366 | 0 |
| PPYTVVYFPVR | 1339.02 | 2 | 4.32 | 0.445 | 0 |
| MLLADQGQSWK | 1276.92 | 2 | 3.18 | 0.235 | 5.96 |
| ASCLYGQLPK | 1079.78 | 2 | 2.47 | 0.165 | 3.23 |
| DQQEAALVDM*VNDGVEDLR | 2134.81 | 3 | 4.86 | 0.437 | 0 |
| DQQEAALVDM*VNDGVEDLR | 2134.8 | 2 | 7.69 | 0.591 | 11.6 |
| TLGLYGK | 751.27 | 1 | 1.84 | 0.222 | 1.06 |

*Oxidation of Methionine (+16 amu)

M**PPYTVVYFPVR**GRCAALR**MLLADQGQSWKEEVVTVETWQEGSLKASCLYGQLPKFQDGDLTLYQSNTILR**HLGR**TLGLYGKDQQEAALVDMVNDGVEDLR**CKYISLIYTNYEAGKDDYVK**ALPGQLKPFETLLSQNQGGK**TFIVGDQISFADYNLLDLLLIHEVLAPGCLDAFPLLSAYVGRLSARPKLK**AFLASPEYVNLPINGNGK**Q

A total of fourteen high-scoring peptides as described in Materials and Methods were identified covering 60% (bold) of the primary sequence of GSTπ protein.

| **Peptide Sequence** | **[M+H]+** | **Charge State** | **Sequest Xcorr** | **Sequest dCn** | **X!Tandem –log(e) value** |
| --- | --- | --- | --- | --- | --- |
| SGGTTMYPGIADR | 1,325.62 | 2 | 2.53 | 0.34 | 3.8 |
| EKMTQIMFETF | 1,404.65 | 2 | 3.13 | 0.363 | 3.92 |
| SFTTTAEREIVR | 1,409.74 | 2 | 2.78 | 0.302 | 0 |
| MQKEITALAPSTMK | 1,548.81 | 2 | 4.66 | 0.319 | 5.39 |
| MQKEITALAPSTM*K | 1,564.81 | 2 | 3.42 | 0.345 | 2.19 |
| FRC*PEALFQPSFL | 1,611.80 | 2 | 3.04 | 0.365 | 0 |
| LDLAGRDLTDYLMK | 1,623.84 | 2 | 2.7 | 0.143 | 2.54 |
| GYSFTTTAEREIVR | 1,629.82 | 2 | 2.92 | 0.164 | 0 |
| GYSFTTTAEREIVR | 1,629.82 | 2 | 3.21 | 0.193 | 0 |
| TVLSGGTTMYPGIADR | 1,638.82 | 2 | 3.84 | 0.431 | 4.96 |
| LDLAGRDLTDYLM*K | 1,639.84 | 2 | 2.5 | 0.291 | 3.48 |
| LDLAGRDLTDYLM*K | 1,639.84 | 2 | 2.66 | 0.316 | 1.72 |
| SYELPDGQVITIGNER | 1,790.89 | 2 | 4.11 | 0.368 | 5.66 |
| ANTVLSGGTTMYPGIADR | 1,823.90 | 2 | 3.94 | 0.478 | 5.05 |
| EKLCYVALDFEQEMATAASSSSLEK | 2,750.29 | 3 | 0 | 0 | 2.68 |
| ANTVLSGGTTM*YPGIADR | 1,839.89 | 2 | 5.08 | 0.526 | 0 |
| VAPEEHPVLLTEAPLNPK | 1,954.07 | 2 | 3.41 | 0.374 | 5.43 |
| VAPEEHPVLLTEAPLNPK | 1,954.07 | 2 | 4.33 | 0.364 | 5.09 |
| DLYANTVLSGGTTMYPGIADR | 2,215.07 | 2 | 4.51 | 0.538 | 5.68 |
| DLYANTVLSGGTTMYPGIADR | 2,215.07 | 2 | 3.97 | 0.462 | 5.72 |
| DLYANTVLSGGTTM*YPGIADR | 2,231.07 | 2 | 4.24 | 0.438 | 5.74 |
| TTGIVMDSGDGVTHTVPIYEGY | 2,312.08 | 2 | 3.45 | 0.468 | 3.8 |
| TTGIVMDSGDGVTHTVPIYEGY | 2,312.08 | 2 | 3.11 | 0.4 | 5.46 |
| TTGIVM*DSGDGVTHTVPIYEGY | 2,328.07 | 2 | 2.92 | 0.357 | 0 |
| TTGIVM*DSGDGVTHTVPIYEGY | 2,328.07 | 2 | 3.19 | 0.388 | 0 |
| LC*YVALDFEQEMATAASSSSLEK | 2,550.17 | 2 | 4.88 | 0.464 | 6.96 |
| LC*YVALDFEQEM*ATAASSSSLEK | 2,566.17 | 2 | 5.4 | 0.558 | 8.16 |

*Oxidation of Methionine (+16 amu)

*Carbamidomethylation of Cysteine (+57 amu)

MDDDIAALVVDNGSGMCK**AGFAGDDAPR**AVFPSIVGRPRHQGVMVGMGQKDSYVGDEAQSKRGILTLKYPIEHGIVTNWDDMEKIWHHTFYNELR**VAPEEHPVLLTEAPLNPK**ANR**EKMTQIMFETF**NTPAMYVAIQAVLSLYASGR**TTGIVMDSGDGVTHTVPIYEGY**ALPHAILR**LDLAGRDLTDYLMK**ILTER**GYSFTTTAEREIVR**DIK**EKLCYVALDFEQEMATAASSSSLEKSYELPDGQVITIGNERFRCPEALFQPSFL**GMESCGIHETTFNSIMKCDVDIRK**DLYANTVLSGGTTMYPGIADRMQKEITALAPSTMK**IKIIAPPERKYSVWIGGSILASLSTFQQMWISKQEYDESGPSIVHRKCF

A total of fifteen high-scoring peptides as described in Materials and Methods were identified covering 47% (bold) of the primary sequence of actin I protein.
